# Hearing loss and its associated factors among metal workshop workers at Gondar city, Northwest Ethiopia

**DOI:** 10.3389/fpubh.2022.919239

**Published:** 2022-08-08

**Authors:** Mihret Melese, Dagnew Getnet Adugna, Bezawit Mulat, Ayechew Adera

**Affiliations:** ^1^Department of Human Physiology, College of Medicine and Health Sciences, University of Gondar, Gondar, Ethiopia; ^2^Department of Human Anatomy, College of Medicine and Health Sciences, University of Gondar, Gondar, Ethiopia

**Keywords:** hearing loss, metalworkers, associated factors, Gondar city, audiometer

## Abstract

**Introduction:**

Noise-induced hearing loss is a permanent sensorineural deficiency, which is caused by exposure to excessive noise sound. Although noise-induced hearing loss due to industrialization is a main public health problem in Ethiopia, studies on the prevalence and associated factors of hearing loss are scarce.

**Objectives:**

This study aimed to assess the prevalence and associated factors of hearing loss among workers at a metal workshop in Gondar city, Northwest Ethiopia.

**Methods:**

A cross-sectional study was employed among 300 participants using a stratified sampling technique. Data were collected using an interviewer-administered questionnaire. Bivariable and multivariable logistic regressions were conducted. In the multivariable logistic regression model, adjusted odds ratios (AOR) with a 95% confidence interval (CI) and a *p* < 0.05 were computed to determine the level of significance.

**Results:**

The prevalence of hearing loss among metal workshop workers was 30.7% [95% CI: (25.7, 35.7)]. Age between 30 and 44 years [AOR = 2.9; 95% CI: 1.2, 7.1], age between 45 and 65 years [AOR = 3.8; 95% CI (1.5, 9.5)], cigarette smoking [AOR = 2.3; 95% CI: 1.2, 4.5], working area noise level >85 dB [AOR = 2.2; 95% CI: 1.1, 6.5], working experience of 6–10 years [AOR = 1.8; 95% CI: 1.4, 6.0], working experience >10 years [AOR = 3.5; 95% CI: 1.3, 4.3], and using ear protection devices [AOR = 0.3; 95% CI: 0.1, 0.6] were significantly associated with hearing loss.

**Conclusion:**

The prevalence of hearing loss was considerably high. This study revealed that advanced age, cigarette smoking, increased working area noise level, and working experiences were found to increase the odds of having hearing loss. Therefore, it is important to emphasize metal workshop workers that are at high risk of hearing loss and develop preventive strategies to reduce the burden of this problem. Besides, minimizing working area noise levels, proper utilization of ear protection devices, and creating awareness about the impact of hearing loss are recommended.

## Introduction

Hearing loss is defined as if an individual has a threshold level of ≥25 dBA at the frequencies 250, 500, 1,000, 2,000, 4,000, and 8,000 Hz (1). Noise-induced hearing loss (NIHL) is a permanent sensorineural deficiency, which is caused by exposure to excessive noise sound (2). Hearing impairment due to occupational noise exposure is an important public health concern throughout the world (3, 4). Indeed, exposure to various sound sources including metal industries, nightclubs, bars, cinemas, concerts, and live sporting events increases the likelihood of developing hearing loss (5).

Previous studies have confirmed that exposure to a noise level of more than 85 dB can lead to an increased risk of hearing loss (6). Interestingly, mechanical damage to the cochlea is the main pathological change, which occurs as the result of the high intensity of noise. The hair cells in the organ of Corti are directly affected by the high intensity of continuous sound causing the constriction of cochlear blood vessels. This leads to a decrease in the flow of blood to the cochlea and causes ischemia and hypoxia of hair cells (7, 8).

Globally, about 16% of disabling hearing loss in adults is due to occupational-related noise (3). Studies conducted in the United States revealed that about 33% of workers are potentially affected by occupational noise-induced hearing loss (9). In Canada, 35% of metal workshop workers experienced noise-induced hearing loss (10) and in Japan, 61.5% of participants experienced this problem (11). Noise-induced hearing loss due to excessive noise levels was high in different countries, for instance, it was 30.4% in Nepal (12), 35.0% in Jordan (13), and 38% in a study conducted in Saudi Arabia (14). Noise-induced hearing loss in Botswana accounts for 78% of all metal workshop workers (15). In Sudan, about 62.5, 10, and 12.5% of metal workers develop noise-induced hearing loss in the bilateral ear, right, and left ear, respectively (16). Hearing loss is the 4th main root cause of disability, with an estimated yearly cost of ≥750 billion dollars (17).

Workplace noise exposure shows a significant financial and health impact on individuals and society levels (18, 19). It has also a major psychosocial impact on individuals' daily life. Furthermore, several studies showed that individuals with hearing loss are prone to social isolation, impaired communication with coworkers and family, decreased ability to monitor the work environment, decreased self-esteem, and loss of productivity (16, 20).

Based on previous studies, factors that are associated with hearing loss include occupational noise in the workplace (21), duration of exposure and intensity of working area noise level (22), cigarette smoking (23), age (24), the use of ototoxic medicines (e.g., aminoglycosides), head injury, and chronic ear infection (25, 26). Different studies revealed that minimizing working area noise levels, use of ear protective devices, and creating awareness about the consequence of hearing loss for employers were important means to minimize the risk of acquiring occupational noise-induced hearing loss (3, 27).

Nowadays, hearing loss due to industrialization is a main public health problem in Sub-Saharan Africa, particularly in Ethiopia (12). Although the number of metal workshops in Ethiopia have increased to meet the rising demand for different infrastructures, the level of occupational noise exposure is still not clear. Besides, studies showing the prevalence of hearing loss among metal workshop workers are scarce. Therefore, this study aimed to determine the prevalence of hearing loss and its associated factors among metal workers in Gondar city.

## Materials and methods

### Study setting, design, and period

A cross-sectional study was conducted among workers at a metal workshop in Gondar city from March to May 2021. Gondar is one of the historical cities in the country and is located in the Central Gondar Zone of the Amhara National Regional State. It is far around 750 km from Addis Ababa, the capital city of Ethiopia. Based on the Gondar city trade and industry office report, a total of 409 employees are found in 46 metal workshops.

### Population

All metal factory workers at Gondar city were the source of population. All metal workshop workers who are presented in the study area during the study period were included in the study population.

### Eligibility criteria

All adult metal workshop workers whose age is ≥18 years and working in the metal factory at least for 6 months were eligible to participate in the study.

### Sample size determination and sampling procedure

The sample size of the study was determined using a single population proportion formula by considering: the confidence level (95%), the margin of error = 5%, and the prevalence of hearing loss = 28.2% taken from the previous study done in Nigeria (28) since no previous similar study done in Ethiopia. By adding a 5% non-response rate, the required sample size was 328. A stratified random sampling technique was performed to select the study participants. All metal workshops were stratified by the working area noise level. A total of 328 metal workshop workers were selected by using a simple random technique in all-metal workshops after proportion allocation was made.

### Study variables

The dependent variable *for this study was* hearing loss (yes/no). Socio-demographic factors such as sex, age, educational status, marital status, and monthly income were assessed. Behavioral and working area-related behaviors like cigarette smoking, alcohol intake, utilization of hearing protection devices (earmuff, and earplugs), work experience, duration of exposure, the intensity of exposure, leisure-time noise exposure, and working area noise level were also assessed.

### Operational definitions

#### Metal workshop workers

Those workers who were making metal welding, cutting, and reshaping to create useful objects.

#### Hearing loss

It was diagnosed if an individual has a threshold level of ≥25 dBA at the frequencies 250, 500, 1,000, 2,000, 4,000, and 8,000 Hz as measured by an audiometer test (5).

#### Working area noise levels

Workers who are exposed to ≥85 dB are at higher risk of developing hearing loss.

#### Cigarette smoking

Metal workshop workers who reported smoking at least for 6 months.

#### Alcohol consumption

Participants who drunk any alcohol at least 1 time per day for 1 year.

### Data collection tools and procedures

Data on socio-demographic and behavioral factors like age, sex, marital status, educational status, income, cigarette smoking, and alcohol consumption were collected by interviewing the metal workshop workers using structured interviewer-administered questionnaires. The questionnaire was initially developed in English, translated into the local language (Amharic), and then translated back into English by experts who are fluent in both languages. Participants were interviewed by four Bsc nurses under the supervision of a principal investigator. Two types of instruments used for measuring sound levels were the sound level meter (model, SL-5868I) (in the recording of the noise levels in several metal workshops) and the pure-tone audiometer (to evaluate the hearing threshold of participants).

#### Physical measurements

##### Working area noise level measurement

The environment noise (working area noise level) was measured in work stations using the sound level meter (model, SL-5868I) with a measurement range between 25 and 130 dB. In this study, the sound level meter was calibrated before and after each use and workplace noise level measurements were taken on slow response. The device was placed at an approximate distance of at least 1 m from the noise source. The audiologist holds the device by facing the microphone toward the noise source and observed the measurement on the liquid crystal display. The working area noise level was measured as an average value of 5 measurements hourly throughout working times for 8 h since the production process was inconsistent, and 8-h time-weighted average was taken. Lastly, the mean working area noise level of 8-h time-weighted average on five different days was taken for each working area (29).

##### Audiometric measurements

Pure-tone audiometer calibration was done daily using supposedly normal people with normal hearing prior to any audiological evaluation of the participants. All audiometric tests were done in a quiet room with a background noise level of 36–40 dB before the workers entered their work stations to avoid the effects of temporary threshold shifts, due to continuous noise exposure inside the working area. Participants were advised of a planned audiometric test, therefore, they can have a “quiet time” or “acoustic rest” of ideally 16 h before the audiometric test (30). The participants were thoroughly instructed about the test and asked to sit still and not to talk. Earphones were placed on the participant's ears. The earphones are connected to the machine that will deliver the tones and different sounds of speech to the participant's ear. Participants were familiarized with the signal before threshold determination by presenting a signal of sufficient intensity to evoke a clear response. The right and left ears of participants were tested consequently by adjusting the audiometer machine. Then, participants were asked to press the pointer of the audiometer when they heard the sound and the audiologist records the hearing threshold level in dB at a frequency of 250, 500, 1,000, 2,000, 4,000, and 8,000 HZ. Three consecutive audiometric measurements were performed before the workers entered their workstations. The average audiometric measurement of the three consecutive records was analyzed. Finally, hearing loss was diagnosed if an individual has a threshold level of ≥25 dBA at the frequencies 250, 500, 1,000, 2,000, 4,000, and 8,000 Hz (5).

##### Anthropometric measurements

The height of metal workshop workers was measured with calibrated height measuring steel attached to the beam balance in a standing upright position with bare feet. Participants' weight was also measured using a calibrated weight scale and recorded accurately to 100 g. Body mass index was calculated based on the result of anthropometric measurements and it was categorized as underweight (<18.5 kg/m^2^), normal (18.5–24.99 kg/m^2^), overweight (25–29.99 kg/m^2^), and obese (≥30 kg/m^2^).

### Data quality control

To ensure data quality, 2-day training was given for data collectors. Pretesting of the questionnaire was done with 5% of metal workshop workers at Dabat town to ensure its validity. Daily close supervision of the data collectors was made by the principal investigator during the data collection period. The data were checked for completeness and consistency before entry.

### Data processing and analysis

After data collection, data were entered into Epi data 4.6 and then exported into Statistical Package for Social Sciences (SPSS) version 25 software for analysis. Descriptive statistics (mean, median, frequency, percentage, interquartile range) were used to summarize the characteristics of the study population through tables and charts. The normality of continuous data was checked by the Shapiro-Wilk test (*p* = 0.001). The Hosmer–Lemeshow test was done to assess model goodness-of-fit (*p* ≥ 0.05). Independent variables having a *p* ≤ 0.2 in bi-variable analyses were included in the multivariable analysis to control confounders in binary logistic regression models. An odds ratio (OR) at a 95% confidence interval (CI) was determined to see the strength of association between the independent variables and outcome variables. Factors with a *p* ≤ 0.05 in the multi-variable regression model were considered statistically significant.

## Results

### Socio-demographic and anthropometric characteristics

A total of 300 metalworkers participated in this study with a response rate of 91.5%. A majority (94.4%) of study participants were males. The median age of the participants was 35 years with an interquartile range of 16. Nearly 47% of study participants were unmarried. Regarding educational status, about one-third (35.3%) of participants were diplomas and above. A majority (92.4%) of the participants had a normal body mass index (Table 1).

**Table 1:** Socio-demographic and anthropometric characteristics of study participants among metal workers in Gondar city, Northwest Ethiopia, 2021.

**Variables**	**Category**	**Frequency**	**Percentage**
Sex	Male	283	94.3
	Female	17	5.7
Age in years	18–29	103	34.4
	30–44	106	35.3
	45–65	91	30.3
Average monthly income (Ethiopian birr)	<1,500	17	6
	1,500–2,000	27	9
	2,001–3,200	120	40
	>3,200	136	45
Marital status	Single	139	46.3
	Married	125	41.7
	Divorced	20	7
	Widow	16	5
Educational levels	Illiterate	18	6
	Primary school	83	27.7
	Secondary school	93	31
	Diploma and above	106	35.3
BMI(kg/m^2^)	<18.5	3	1
	18.5–24.9	277	92.4
	25–29.5	20	6.6

### Behavioral and work-related characteristics

About one-third (34.7%) of the participants were smokers. One-third (34%) of the participants had more than 10 years of working experience. Among the total metal workers, about 57.3% of participants have not used ear protection devices and nearly half (52%) of the participants were exposed to noise levels >85 dB (Table 2).

**Table 2:** Behavioral and work-related characteristics of study participants among metal workers in Gondar city, Northwest Ethiopia, 2021.

**Variables**	**Category**	**Frequency**	**Percentage**
Previous working experience	Military	37	12.3
	Mining	50	16.7
	Construction	15	5
	Garage	46	15.3
	Others^*^	152	50.7
Current working experience (in years)	1–5	107	36
	6–10	90	30
	>10	102	34
Music listing with earphones	Yes	101	33.7
	No	199	66.3
Working area noise level (dB)	<85	143	47.3
	≥85	157	52.7
Alcohol consumption	Yes	117	39
	No	183	61
Smoking cigarette	Yes	107	34.7
	No	193	65.3
Use of ear protection devices	Yes	127	42.7
	No	173	57.3

* Students, merchants, farmers.

### Prevalence of hearing loss among metal workers

**Figure 1:**
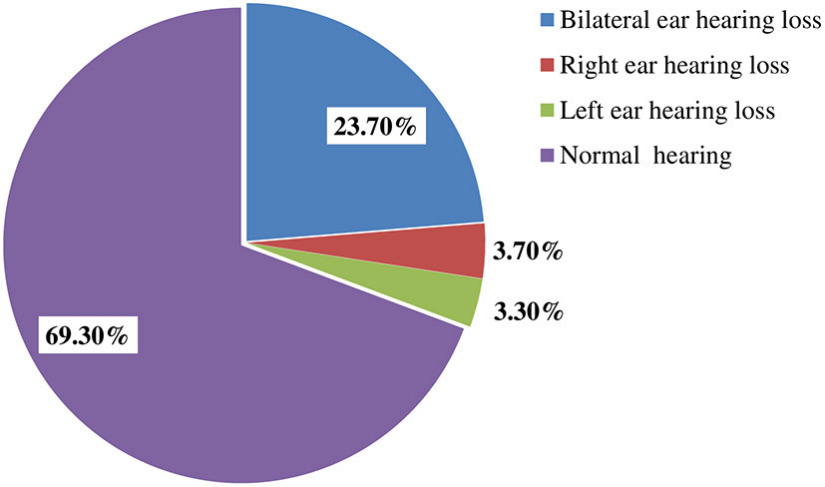
The prevalence of hearing loss according to types among metalworkers in Gondar city, Northwest, Ethiopia.

**Figure 2:**
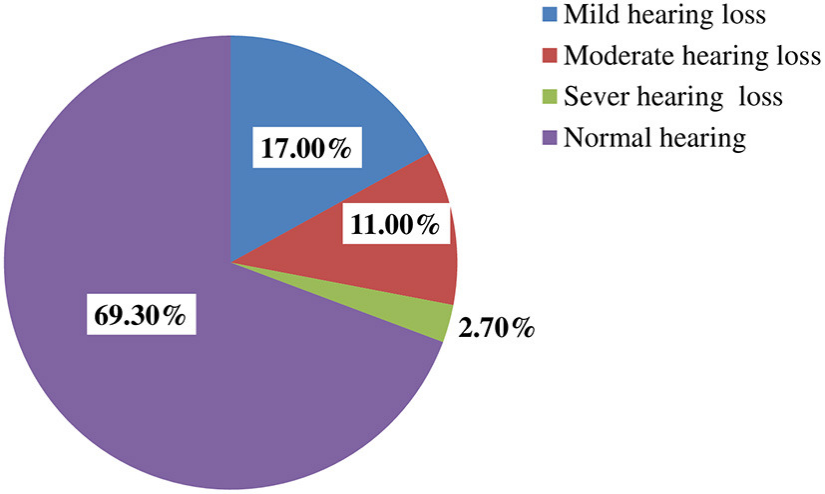
The prevalence of hearing loss according to the degree of severity among metalworkers in Gondar city, Northwest, Ethiopia.

In this study, the prevalence of hearing loss among metalworkers was found to be 30.7% (95% CI: 25.7, 35.7). In addition, 23.7% of participants had bilateral hearing loss, whereas 7% had unilateral hearing loss (3.7% right ear, and 3.3% left ear) (Figure 1). Regarding the degree of hearing loss, about 17, 11, and 2.7% of participants had a mild, moderate, and severe form of hearing loss, respectively (Figure 2).

### Factors associated with hearing loss

In the bivariable analysis: work experience, working area noise level, age, cigarette smoking, alcohol intake, listening to music, and use of ear protection devices were significantly associated with hearing loss. However, in multivariable binary logistic regression analysis: only age, working experience, use of ear protection devices, smoking, and working area noise level were identified as statistically significant risk factors for hearing loss (Table 3).

**Table 3:** Factors associated with hearing loss among metal workshop workers in Gondar city, Northwest Ethiopia, 2021.

**Variables**		**Category**	**Hearing loss**	**COR (95% CI)**	**AOR (95%CI)**
		**Yes**		**No**	
Age in years	18–29	11 (10.7%)	92 (89.3%)	1	1
	30–44	29 (27.4%)	77 (72.6%)	3.2 (1.5, 6.7)	2.9 (1.2, 7.1)^*^
	45–65	52 (52.1%)	39 (42.9%)	11.2(5.3, 23.6)	3.8 (1.5, 9.5)^*^
Current working experience (years)	1–5	12 (13%)	96 (88.9%)	1	1
	6–10	29 (31.5%)	61 (67.8%)	3.8 (1.8, 8.8)	1.8 (1.4, 6.0)^*^
	>10	51 (55.4%)	51 (38.2%)	8.0 (4.1, 18.5)	3.5 (1.3, 4.3)^**^
Music listing with, earphone	Listener	50 (54.3%)	82 (39.4%)	1.8 (1.1, 3.0)	0.8 (0.4, 1.6)
	Non-listener	42 (37.9%)	126 (62.1%)	1	–
Working area noise level (dB)	<85	17 (11.9%)	126 (88.1%)	1	–
	≥85	75 (47.8%)	82 (52.2%)	6.7 (3.7, 12.9)	2.2 (1.1, 6.5)^*^
Cigarette smoking	Smoker	62 (57.9%)	45 (42.1%)	10.6 (4.4, 22)	2.26 (1.1,4.5)^*^
	Non-smoker	30 (15.5%)	163 (84.5%)	1	1
Ear protection devices	Used	16 (12.6%)	111 (87.4%)	0.6 (0.3, 0.9)	0.3 (0.1, 0.6)^*^
	Not used	76 (43.9%)	97 (56.1%)	1	1
Alcohol consumption	Drunker	50 (37%)	85 (63%)	1.72 (1.1, 2.8)	1.23 (0.9, 4.5)
	Non-drunker	42 (25. 5%)	123 (74.5%)	1	1

1= reference category, Hosmer Lemshow = 0.27, ^*^p ≤ 0.05, ^**^p ≤ 0.001.

Metal workshop workers in the age group between 45 and 65 years were 3.8 times more likely to develop hearing loss when compared to those in the age group between 18 and 29 years (AOR = 3.8; 95% CI: 1.5, 9.5). Similarly, the likelihood of hearing loss among metal workshop workers in the age group between 30 and 44 years was 2.9 times higher compared to those in the age group between 18 and 29 years (AOR = 2.9; 95% CI: 1.2, 7.1). Participants who had more than 10 years of working experience were 3.5 times more likely to have a hearing loss than those participants who had 1–5 years (AOR = 3.5; 95% CI: 1.3, 4.3). Participants with working experience of between 6 and 10 years were 1.8 times higher to have hearing loss as compared to those participants between 1 and 5 years [AOR = 1.8; 95% CI: (1.4, 6.0)]. Participants who used ear protection devices were 70% less likely to develop hearing loss as compared to those who did not use ear protection devices (AOR = 0.3; 95% CI: 0.1, 0.6). Metalworkers who were exposed to a working area noise level of more than 85 dB had 2.2-fold higher odds of hearing loss than those who were exposed to <85 dB (AOR = 2.2; 95% CI: 1.1, 6.5). A smoker had a 2.3 times higher chance of developing hearing loss as compared to non-smokers (AOR = 2.3; 95% CI: 1.2, 4.5) (Table 3).

## Discussion

The purpose of the present study was to assess the prevalence of hearing loss among metal workshop workers and its associated factors in Gondar city. In this study, the overall prevalence of hearing loss was 30.7% (95% CI: 25.7, 35.7). This finding is similar to previous studies done in Nepal (30.4%) (12), Rwanda (35%) (31), and Nigeria (26%) (32). This might be due to the similarity of the study design used and the working-related characteristics of participants. However, the values obtained as results of this study are lower than other studies conducted in Malaysia (73.3%) (33), Thailand (40%) (34), Tanzania (48%) (5), and Zimbabwe (37%) (35). The possible reason for this difference might be due to variation in working area noise level, type, and the number of machines used. This finding is higher than previous studies done in Gondar (20.7%) (36) and Addis Ababa, Ethiopia (22%) (37). The possible reason for the difference between the current study and a study conducted in Gondar might be due to the discrepancy in methods used (the use of audiometer tests in our study may have increased the burden of hearing loss) and study population (since the previous studies were conducted among metal and woodwork workers). Besides, the difference between the present study and Addis Ababa could be attributed to the difference in the operational definition of hearing loss (>30 dBA at 4,000 Hz audiometric result in Addis Ababa but ≥25 dBA in our study) and study area. The current finding was also higher than the 2013 WHO global report (15%) (38). This could be due to the variations in noise exposure levels and the implementation of occupational health and safety measures that protect against hearing loss (39).

The present study showed that 23.7% of participants had bilateral hearing loss, whereas 7% had unilateral hearing loss (3.7% right ear and 3.3% left ear). The prevalence of bilateral hearing loss in the current finding is lower than the similar findings of a study done in Sudan (62.5%) (40). In both studies, the right ear was more affected than the left ear. The reason for this could be that most of the study participants were right-handed workers whose right ear is closer to the noise source, and hence, received more sound energy, which leads to the possibility of right ear hearing loss (41). This might be also due to noise shielding in the right ear, unequal recovery after excessive noise sound exposure, and unequal sensitivity of both ears and direction of noise exposure.

In this study, factors such as age, working experience, cigarette smoking, and working area noise level were positively associated with hearing loss. However, the use of ear protection devices was negatively associated with hearing loss. The current study revealed that metal workshop workers in the age group between 30 and 44 years and 45 and 65 years were more likely to develop hearing loss when compared to those in the age group between 18 and 29 years. This finding is consistent with the previous studies (24, 34, 42–44). This could be due to the aging effect, which impacts the cochlea of the inner ear in which self-regeneration ability is impaired. As a result, a loss or damage of hair cells could be irreversible and causes permanent hearing loss (38). Moreover, hearing loss might be associated with the primary degeneration of outer hair cells, spiral ganglion cells, and nerve fibers during aging (45).

The finding of this study indicated that participants with a working experience of 6–10 years and above 10 years had a higher chance of developing hearing loss than those participants between 1 and 5 years. This is supported by previous literature done elsewhere (5, 10, 12, 32, 37). The possible reason could be chronic exposure to noise that causes direct mechanical damage to hair cells in the cochlea of the inner ear, which may lead to the generation of toxic free radicals, and eventually result in necrotic and apoptosis cell death (46, 47). However, a study done in Thailand showed that working experience was not significantly associated with hearing loss (34). The possible explanation for this variation could be attributed to the fact that the majority of participants in Thailand were young laborers with a short period of working experience. Additionally, there is also a variation in the implementation of health and occupational safety measures that protect against hearing loss during working hours (48).

The present study indicated that exposure to working area noise levels was associated with hearing loss. Metalworkers who were exposed to the noise level of ≥85 dB had higher odds of hearing loss than those who were exposed to <85 dB. Similar findings were found in other studies (5, 21, 34, 35, 37, 49–51). This might be due to the direct mechanical damage and degeneration of hairy cells of the organ of Corti *via* excessive sound (38).

According to the current study, participants who used ear protection devices were less likely to develop hearing loss than their counterparts. This is in line with the previous studies done elsewhere (10, 34, 41, 49). This might be due to the protective effect of ear protection devices that minimize the incoming sound reaching the inner ear (34).

Furthermore, the result of this study showed that the odds of having hearing loss were higher among cigarette smokers than non-smokers. This is supported by the previous studies conducted in Brazil, Japan, and Nepal (12, 22, 23, 42). This might be because cigarette burning releases chemicals including toluene, styrene, and xylene, which have the potential to cause an ototoxic effect on hair cells. In addition, carbon monoxide released from burning cigarettes reduces cochlear blood oxygen levels as it makes the dissociation of oxygen from hemoglobin difficult and leads to hair cell hypoxia and degeneration (11).

The strength of this study was that data were collected from various metalworking areas (Multicenter) allowing us to generalize the findings to all metal workshop workers in Gondar city. This study has some limitations: First, the study did not show the cause and effect relationship since it is a cross-sectional study design. Second, the study did not address extra working significant exposure. Furthermore, the study will be a base for future investigators to perform better study designs like prospective cohort and experimental studies in this setting to bring findings with better validity.

## Conclusion

The prevalence of hearing loss among metal workshop workers in Gondar city was relatively high. The study indicated that advanced age, cigarette smoking, high working area noise level, and prolonged working experience were found to increase the odds of having hearing loss among metalworkers. Therefore, it is important to emphasize metal workshop workers that are at high risk of hearing loss and develop preventive strategies to reduce the burden of this problem. This study recommends the proper utilization of ear protection devices. Besides minimizing working area noise levels, proper utilization of ear protection devices and creating awareness about the impact of hearing loss are also recommended.

## Data availability statement

The raw data supporting the conclusions of this article will be made available by the authors, without undue reservation.

## Ethics statement

The studies involving human participants were reviewed and approved by School of Medicine Ethical Review Committee, College of Medicine and Health Sciences, University of Gondar. The patients/participants provided their written informed consent to participate in this study.

## Author contributions

DA and MM conceived and designed the study, participated in the data collection process, analyzed data, and wrote the manuscript. BM and AA participated in data analysis, drafting of the manuscript, and advising the whole research paper and also were involved in the interpretation of the data and contributed to manuscript preparation. All authors read and approved the final manuscript.

## Conflict of interest

The authors declare that the research was conducted in the absence of any commercial or financial relationships that could be construed as a potential conflict of interest. The reviewer BF declared a shared affiliation with the authors DA, MM, AA, and BM to the handling editor at the time of review.

## Publisher's note

All claims expressed in this article are solely those of the authors and do not necessarily represent those of their affiliated organizations, or those of the publisher, the editors and the reviewers. Any product that may be evaluated in this article, or claim that may be made by its manufacturer, is not guaranteed or endorsed by the publisher.
